# PCR detection of *Anaplasma phagocytophilum* in goat flocks in an area endemic for tick-borne fever in Switzerland

**DOI:** 10.1051/parasite/2011181057

**Published:** 2011-02-15

**Authors:** C. Silaghi, M.C. Scheuerle, L.M. Friche Passos, C. Thiel, K. Pfister

**Affiliations:** 1 Institute of Comparative Tropical Medicine and Parasitology, Faculty of Veterinary Medicine, Ludwig-Maximilians-University Leopoldstr. 5 80802 Munich Germany; 2 Universidade Federal de Minas Gerais Brazil

**Keywords:** *Anaplasma phagocytophilum*, tick-borne fever, goat, cattle, PCR, *16S rRNA* gene, *groEL* gene, *msp4* gene, Switzerland, *Anaplasma phagocytophilum*, fièvre récurrente à tiques, chèvre, bovin, PCR, gène *16S rRNA*, gène *groEL*, gène *msp4*, Suisse

## Abstract

Central Switzerland is a highly endemic region for tick-borne fever (TBF) in cattle, however, little is known about *A. phagocytophilum* in goats. In the present study, 72 animals from six goat flocks (373 EDTA blood-samples) in Central Switzerland were analysed for *A. phagocytophilum* DNA. A real-time PCR targeting the msp2 gene of *A. phagocytophilum* was performed and in positive samples the partial *16S rRNA*, *groEL* and *msp4* gene were amplified for sequence analysis. Four DNA extracts were positive. Different sequence types on basis of the amplified genes were found. For comparison, sequences of *A. phagocytophilum* from 12 cattle (originating from Switzerland and Southern Germany) were analysed. The *16S rRNA* gene sequences from cattle were all identical amongst each other, but the *groEL* and *msp4* gene differed depending on the origin of the cattle samples and differed from the variants from goats. This study clearly provides molecular evidence for the presence of different types of *A. phagocytophilum* in goat flocks in Switzerland, a fact which deserves more thorough attention in clinical studies.

## Abstract (fr)

La Suisse centrale est une région hautement endémique de fièvre récurrente à tiques du bétail, mais le rôle d’*A. phagocytophilum* chez les chèvres n’est pas vraiment connu. Le sang de 72 chèvres provenant de six troupeaux de la Suisse centrale a été analysé (373 échantillons de sang EDTA au total) dans le but de détecter la présence de l’ADN d’*A. phagocytophilum*. Une réaction PCR en temps réel ciblant le gène msp2 d’*A. phagocytophilum* a été réalisée. Dans les échantillons positifs, les gènes partiels *16S rRNA*, *groEL* et *msp4* ont été amplifiés afin d’en analyser les séquences. Quatre extraits d’ADN étaient positifs. Différents types de séquences sur la base des gènes amplifiés ont été identifiés. Dans un but de comparaison, des séquences d’*A. phagocytophilum* trouvés chez 12 bovins originaires d’Allemagne du Sud et de Suisse ont été analysées. Les séquences du gène *16S rRNA* étaient toutes identiques quel que soit le bétail, mais les gènes *groEL* et *msp4* étaient variables selon l’origine animale des échantillons, mais également selon l’origine des chèvres. Cette étude apporte l’évidence moléculaire de la présence de différents types d’*A. phagocytophilum* dans les troupeaux de chèvres en Suisse, un résultat qui mérite plus d’attention pour de futures études

## Introduction

*Anaplasma (A.) phagocytophilum* is an obligate tick-transmitted intracellular bacterium. It causes granulocytic anaplasmosis in dogs, cats, horses and humans and tick-borne fever (TBF) in ruminants (Rikihisa, 1991). TBF in cattle is characterized by high fever, respiratory symptoms, abortion, a sudden decrease in milk production, leucopenia and can cause severe economic losses in ruminants (Woldehiwet, 2006; Stuen, 2007). However, symptoms may vary depending on the infected species, the variant of *A. phagocytophilum* involved or the age, condition and immune status of the host (Woldehiwet, 2006; Stuen, 2007). *A. phagocytophilum* can show great genetic heterogeneity between different geographic regions or host species (reviewed in Woldehiwet, 2006). In sheep flocks, different genetic variants of *A. phagocytophilum* may circulate within one flock (Ladbury *et al.*, 2008).

**Fig 1.**
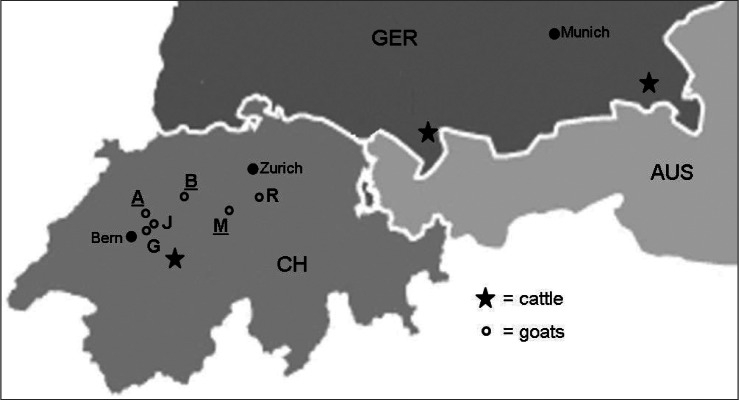
Origin of the cattle and goat EDTA-blood samples (May to October 2008), which were investigated for *Anaplasma phagocytophilum* by real-time PCR. A, B, G, J, M, R, goat flocks investigated; underlined letter = flocks where *A. phagocytophilum* was found. AUS, Austria; CH, Switzerland; GER, Germany.

In Switzerland, the first report of TBF in cattle was from the “Bernese Oberland” (Pfister *et al.*, 1987) (Fig. 1) and further evidence of *A. phagocytophilum* in cattle in Switzerland was given later (Hofmann- Lehmann et al., 2004). Even though there have been isolated reports on outbreaks of TBF in domestic and free-living feral goats, very little is known about the general presence of *A. phagocytophilum* in goats (Foster & Greig, 1969; Gray *et al.*, 1988). Experimental infections of sheep and goats with *A. phagocytophilum* showed that all animals reacted with fever, rickettsiaemia, lymphocytopenia and reduced packed cell volume (PCV). However, fever in sheep set in earlier and lasted longer than in goats (Gokce & Woldehiwet, 1999). The main vector for *A. phagocytophilum* in central Europe is *Ixodes (I.) ricinus* (Linné 1758), which is the most abundant tick, also in Switzerland. There, PCR prevalence with *A. phagocytophilum* in *I. ricinus* ranges from 0.5 to 2.2% (Leutenegger *et al.*, 1999; Pusterla et al., 1999b; Liz *et al.*, 2000; Wicki *et al.*, 2000).

In the present study, goat flocks in Central Switzerland were investigated with real-time PCR for the presence of *A. phagocytophilum*. In positive samples, sequence analysis was performed for partial *16S rRNA*, *groEL* and *msp4* gene, comparing *A. phagocytophilum* appearing in small and large ruminants.

## Materials and Methods

During a study on the implementation of the FAMACHA© test in goats suffering from gastrointestinal nematodes (GIN), faecal samples and EDTA-blood samples for determination of PCV value, were taken monthly from May to October 2008 from six goat flocks from Central Switzerland (Scheuerle *et al.*, 2010). The EDTA-blood samples were kept frozen at -26 °C and were subsequently used in this study for the detection of *A. phagocytophilum* DNA in goats. Due to the frozen state of the samples, it was not possible to use them for serological examination.

The six goat flocks (named A, B, G, J, M, R, according to the first letter of the name of their owners) were situated in the Cantons of Bern, Luzern and Zug (Fig. 1). Their pastures (no stream or wetland; altitude 500-1,100 m above sea level) were situated on steep hills surrounded by woodland, and hence were potential tick habitats. *I. ricinus* ticks had been identified previously by exemplary flagging close to the study sites. All animals spent the entire sampling period on the pastures. Each flock consisted of at least one buck, 10 to 100 does with ages ranging from one to 13 years and a lamb group. From each flock, eight to 13 animals were included in the study on GIN and subsequently for this investigation on *A. phagocytophilum*. For flock R in the month of July, blood samples were not available. Overall 373 samples from 72 individuals were tested.

To compare the sequence variants involved, frozen EDTA-blood samples from 12 *A. phagocytophilum*positive cattle were used. The TBF in cattle had been diagnosed positive previously by detection of morulae in the neutrophilic granulocytes in Giemsa-stained blood smears and the blood had been kept frozen. Altogether there were samples from nine cattle (2005), with typical symptoms of clinical TBF (apathy, high fever, sudden decrease in milk production), and three cattle from Southern Germany (2002), with unknown disease status. The Swiss cattle were from the region south of Interlaken, Switzerland and the German cattle from prealpine regions in Germany (Fig. 1). This region in Switzerland is highly endemic for TBF in cattle (P.H. Boss, personal communication).

DNA extraction was carried out with the High Pure PCR template preparation kit (Roche, Mannheim, Germany) according to the manufacturer’s instruction. Quality and quantity of extracts was tested with a spectrophotometer (NanoDrop® ND-1000, PeqLab, Erlangen, Germany). A real-time PCR targeting the *msp2* gene of *A. phagocytophilum* was performed in an iCycler iQ (Bio-Rad, Munich, Germany) as described previously (Courtney *et al.*, 2004; Silaghi *et al.*, 2008). PCR-grade water served as negative, DNA-extracts of *A. phagocytophilum*-positive ticks as positive controls. For positive samples, further PCRs were carried out: a nested PCR targeting a part (530 bp) of the *16S rRNA* gene (Massung *et al.*, 1998) was performed in a GeneAmp 9700 thermocycler (Applied Biosystems, Darmstadt, Germany) using primers ge3a (5’-CACATGCAAGTCGAACGGATTATTC-3’) and ge10r (5’-TTCCGTTAAGAAGGATCTAATCTCC-3’) in the first reaction and primers ge9f (5’-AACGGATTATTCTTTATAGCTTGCT- 3’) and ge2 (5’-GGCAGTATTAAAAGCAGCTCCAGG- 3) in the second amplification. The cycling conditions involved activation of the enzyme at 95 °C for 15 min, 40 cycles (94 °C 30 sec, 55 °C 30 sec, 72 °C 1 min) and a final extension at 72 °C for 5 min. Nested amplification used 1 μl of primary PCR product for 25 cycles. A *groEL* gene hemi-nested PCR assay (Alberti *et al.*, 2005) was carried out with initial activation (15 min 95 °C), 35 cycles (95 °C 60 sec, 62 °C 60 sec, 72 °C 90 sec), and final extension (10 min 72 °C) using primer pair EphplgroEL-F (5’- ATGGTATGCAGTTTGATCGC-3’) and EphplgroEL-R (5’-TCTACTCTGTCTTTGCGTTC-3’) in the first reaction and EphplgroEL-F and EphgroEL-R (5’-TTGAGTACAGCAACACCACCGGAA- 3’) in the second amplification.

A partial *msp4* gene nested PCR was carried out using an approach developed by Bown *et al.* (2007), using primers MSP4AP5 (5’-ATGAATTACAGAGAATTGCTTGTAGG- 3’) and MSP4AP3 (5’-TTAATTGAAAGCAAATCTTGCTCCTATG- 3’) (de la Fuente *et al.*, 2005) followed by a second nested PCR amplification using primers msp4f (5’-CTATTGGYGGNGCYAGAGT-3’) and msp4r (5’-GTTCATCGAAAATTCCGTGGTA-3’) (Bown *et al.*, 2007). Cycling conditions were 15 min at 95 °C, 40 cycles (30 sec 94 °C, 45 sec 54 °C, 1 min 72 °C) and final extension 10 min at 72 °C. Both PCRs were carried out in an Eppendorf Mastercycler gradient (Eppendorf, Hamburg, Germany). The HotStarTaq Polymerase Kit (Qiagen, Hilden, Germany) was used for all PCR experiments in a 50 μl reaction volume.

PCR products were visualized under UV light after 2% Agarose-Gel-Electrophoresis and staining with GelRed® (Biotium, Hayward, USA). After purification with QIAquick PCR Purification Kit according to the manufacturer’s instruction (Qiagen, Hilden, Germany), PCR products were sequenced with forward and reverse primers (Eurofins, Martinsried, Germany). The results were evaluated with Chromas© Lite (www. technelysium.com.au), sequence homology searches were made by BLASTn analysis of GenBank (www. ncbi.nlm.nih.gov) and multiple alignments performed with ClustalW (www.ebi.ac.uk).

## Results

In general, the same goats were sampled over the entire period, for variations in the number of samples see Table 1. Altogether, four of the 373 samples were positive for *A. phagocytophilum* in the real-time PCR. Four different goats were positive and each goat was positive only once. Positive goats were found in three of the six flocks (Fig. 1, Table 1). The age of the positive goats ranged from one to five years; one was of unknown age. All positive goats were females. The PCV of the positive goats ranged from 23 to 35%, the physiological range is from 28 to 40% (Kraft & Dürr, 1999).

**Table 1.** EDTA-blood samples of goats from Central Switzerland, 2008, positive for *Anaplasma phagocytophilum* in real-time PCR (*msp2* gene).

	Month positive goats/total goats tested					
Flock	May	June	July	August	September	October
A	0/11	0/11	1/11	0/16	0/12	0/11
B	0/11	2/11	0/7	0/11	0/12	0/12
G	0/10	0/13	0/13	0/13	0/13	0/12
J	0/7	0/8	0/8	0/8	0/8	0/7
M	0/11	0/11	0/11	0/11	0/11	1/11
R	0/10	0/10	not available	0/11	0/10	0/9

Total	0/60	2/64	1/50	0/70	0/66	1/62

Three different *16S rRNA* gene sequence types differing in up to four nucleotide positions from each other were shown (Table 2). In flock B, two different sequence types were identified within the same month. One sequence was identical to an *A. phagocytophilum* strain from ticks in Sweden and Germany (GenBank accession nos. AJ242783 and AF136714). The other nucleotide sequence obtained in flock B was also identified in flock M (Table 2), identical with another one already obtained from ticks in Sweden and Germany, and also from roe deer in the Czech Republic and humans in Canada (GenBank accession nos. AJ242784, EU839847, AF311343 and AF136713). The sequence type identified in flock A was not 100% identical with sequences in the GenBank database. Differences between *16S rRNA* gene sequences from the present study and selected sequences from the GenBank are shown in Table 2. All three differed, for example, in up to three nucleotide positions from the sequence obtained from cattle with TBF from Switzerland, from cattle in Southern Germany and from variants previously shown to be pathogenic for sheep. All cattle were infected with the same *16S rRNA* gene variant, regardless of the geographic origin. The sequences obtained in this study were deposited at GenBank with accession nos. FJ538288 to FJ538291.

**Table 2.** Comparison of the 526 bp sequences of the *16S rRNA* gene of *Anaplasma phagocytophilum* from this study with selected sequences from the GenBank database.

	Nucleotide position^a^													
Accession no.	64	76	84	170	175	376	46133	463	464	465	467	468	Host	Country
U02521	A	A	G	C	C	G	C	T	G	C	A	G	Human	USA
FJ538288^b^	•	G	A	•	•	•	•	•	•	•	•	•	Goat	CH^f^
FJ538289^c^	•	G	•	•	•	•	•	•	•	•	•	•	Goat	CH
FJ538290^d^	•	•	•	T	•	A	•	•	•	•	•	•	Goat	CH
FJ538291	•	•	A	•	•	•	•	•	•	•	•	•	Cattle^e^	CH, GER^g^
M73220	•	•	A	•	•	•	•	•	•	•	•	•	Sheep	N^h^
AF336220	G	•	A	•	•	•	G	G	A	T	C	C	Sheep	N
AF084907	•	•	•	•	•	•	•	•	•	•	•	•	*I. ricinus*	CH
AF384213	•	G	A	•	•	•	•	•	•	•	•	•	Roe deer	CH
AF384214	•	G	A	•	A	•	•	•	•	•	•	•	Roe deer	CH

a Nucleotide position in relation to the published prototype sequence of the HGA agent (U02521). Comparison was made for bp 59-584 of the prototype with the obtained 526 bp sequence from this study. Dots show identities with the prototype sequence;

b nuclotide sequence from goats B10 (June), M8 (October);

c nucleotide sequence from B11 (June);

d nucleotide sequence from A8 (July);

e identical sequence obtained from 13 cattle;

f CH, Switzerland;

g GER, Germany;

h N, Norway.

The 530 bp partial *groEL* gene was amplified in the four goats and 12 cattle (GenBank accession nos. GQ452225 to GQ452232). Three caprine variants differed in two nucleotide positions from each other and in 17 nucleotide positions from the fourth variant, which differed in only up to two positions from the variants of bovine origin. Altogether four different *groEL* variants of bovine origin were detected. All from Switzerland were the same, whereas the three from Germany were different amongst each other and differed from the Swiss one.

Altogether seven different *msp4* partial gene variants with differences in a total of 14 nucleotide positions were found. Three different variants were found in the amplified part of the *msp4* gene of the goats and four variants were found in the cattle. All Swiss cattle had the same variant, whereas the three German cattle had three different variants. All variants of bovine origin differed from the goat variants (Genbank accession nos. HM028674, HM028675, HM028676, HM028677, HM028678, HM028679, HM028680)

## Discussion

The results show the molecular presence of *A. phagocytophilum* in goats in Central Switzerland. This adds to the knowledge on the epidemiology of *A. phagocytophilum* in Switzerland, which has been described in the tick *I. ricinus* (Leutenegger *et al.*, 1999; Pusterla et al., 1999b; Liz *et al.*, 2000; Wicki *et al.*, 2000) and domestic animals like cattle, dogs and horses (Pfister *et al.*, 1987; Pusterla et al., 1997a; Pusterla et al., 1997b; Pusterla et al., 1998a; Hofmann-Lehmann *et al.*, 2004). Serologic evidence has additionally been given in dogs, cattle, red foxes and chamois (Pusterla et al., 1997c; Pusterla et al., 1998b; Pusterla et al., 1999a; Pusterla et al., 1999b; Liz *et al.*, 2002) and also humans (Pusterla et al., 1998c; Weber *et al.*, 2000). Serological or PCR presence of *A. phagocytophilum* has been described in goats in other countries as well, like Cyprus, Spain and the US (Massung *et al.*, 1998; Amusategui *et al.*, 2006; Chochlakis *et al.*, 2009).

None of the owners of the goat flocks observed any symptoms that could be attributed to *A. phagocytophilum*. However, symptoms of TBF can be very unspecific and the infections may also have been subclinical. The PCR presence of *A. phagocytophilum* without the development of clinical symptoms has been reported in dogs (Jensen *et al.*, 2007, Galke *et al.*, 2009). Therefore infection in the goats may have been unnoticed so far in the region, despite the endemic presence of *A. phagocytophilum* in ticks (Pusterla et al., 1999b) and in cattle (Pfister *et al.*, 1987). Tickinfestation of the goats was very rarely observed by the owners. However, ticks are easily overlooked by farmers, as a regular examination of goats for ticks does not occur.

In Norway, a flock of lambs showed reduced weight gain in connection with subclinical *A. phagocytophilum* infection, even though no apparent tick infestation on the pastures was observed (Stuen *et al.*, 2002). Goats in the present study were massively infested with gastrointestinal nematodes (GIN) (Scheuerle *et al.*, 2010) and therefore, reduced weight gain or any other clinical signs due to *A. phagocytophilum* could have been concealed. The PCV of the four goats was mostly in the physiological range. Here also, low PCV values could have been influenced by the detected infection with the GIN *Haemonchus contortus* (Scheuerle *et al.*, 2010).

In this study, each positive goat was positive only once. Persistence of *A. phagocytophilum* infection in sheep and cattle has previously been shown, resulting in a cyclic parasitaemia, which has been shown in sheep (Stuen *et al.*, 1998; Stuen *et al.*, 2008).

Furthermore, the incubation period in an experimental infection of cattle with *A. phagocytophilum* is between five and nine days and the parasitaemic phase of neutrophils lasts up to two weeks (Pusterla & Braun, 1997). As samples were taken at monthly intervals, active infection may have been overlooked and the results do not allow conclusion to be drawn for the entire pasture period. The finding of *A. phagocytophilum* DNA in goats is important, as, even though no clinical signs were observed which could typically be connected with TBF, infection with *A. phagocytophilum* is known to increase the susceptibility for secondary infections and could thus contribute to productivity losses. Attention should be paid to keeping goats out of the reach of ticks.

In the investigated goat flocks, three different *16S rRNA* gene variants were identified; one flock showed a mixed infection with two different variants in the same month. All these goat variants differed from strains which have previously been shown to be pathogenic for sheep. They also differed from variants from cattle with TBF from the same region in Switzerland.

All *16S rRNA* gene sequences in cattle were the same, regardless of the country of origin and the development of clinical symptoms. The clinical cases in the Swiss cattle were very severe, whereas in Germany, no case of TBF in cattle has so far been reported. Therefore, it may well be that host specificity is more important than geographic origin. The *groEL* gene was amplified in the four goats and 12 cattle. Three caprine variants from three goats were very similar to each other and the fourth one from the fourth goat was very similar to the bovine ones. Four different *groEL* variants of bovine origin were detected; interestingly, all from Switzerland were the same, whereas the three from Germany differed amongst each other in one nucleotide position. The same was the case for the partial *msp4* gene. Therefore, on the basis of these partial genes, there seems to be both geographic and interspecies variation.

The impact of these sequence variations needs further investigation, especially whether there is any influence on the pathogenicity in cattle or the pathogenic potential of *A. phagocytophilum* in goats. Furthermore, future studies need to be undertaken in monitoring active infections, including also the presence of serum antibodies and clinical parameters in goats.

*Disclosure:* The authors declare that no conflict of interest exists.
